# The Safe Sport Allies bystander training: developing a multi-layered program for youth sport participants and their coaches to prevent harassment and abuse in local sport clubs

**DOI:** 10.3389/fpsyg.2024.1389280

**Published:** 2024-06-19

**Authors:** Karolien Adriaens, Helena Verhelle, Gjalt-Jorn Ygram Peters, Leen Haerens, Tine Vertommen

**Affiliations:** ^1^Safeguarding Sport and Society, Centre of Expertise Care and Well-being, Thomas More University of Applied Sciences, Antwerp, Belgium; ^2^Faculty of Psychology, Open University of the Netherlands, Heerlen, Netherlands; ^3^Department of Movement and Sports Sciences, Faculty of Medicine and Health Sciences, Ghent University, Ghent, Belgium; ^4^Social Epidemiology and Health Policy (SEHPO), University of Antwerp, Antwerp, Belgium

**Keywords:** prevention, education, harassment, abuse, sport, bystander, intervention mapping, efficacy

## Abstract

Harassment and abuse represent a pervasive and critical problem in sport with far-reaching consequences. Survivors’ testimonials underscore the profound and enduring impact of these experiences at individual, interpersonal, organizational and community level. Many of their stories reveal painful inaction from responsible adults in the sport organization, aggravating the harm. Other contributing factors to the harm inflicted include a culture of silence, lack of knowledge and understanding of what constitutes abuse, unawareness of reporting and supporting mechanisms, and fear of potential consequences. While effective bystander interventions have been developed outside the sport context, particularly targeting students in higher education, such initiatives have yet to be extensively adapted and assessed within the sport context. To address this gap, the Safe Sport Allies Erasmus+ collaborative partnership relied on the intervention mapping approach as a guiding framework to systematically develop a bystander training program (i.e., Safe Sport Allies) to train youth sport participants and youth sport coaches to act as effective bystanders. The current paper describes the comprehensive development process and provides an overview of implementation and evaluation possibilities. Throughout the paper, it is explained how each step of the Intervention Mapping approach shaped the Safe Sport Allies bystander training program. The program development, and the developed plans for implementation and evaluation are presented, shedding light on challenges encountered. The bystander training program developed in this paper and the implementation and evaluation plans can serve as an outline to build future interventions within this critical domain of safeguarding in sport.

## 1 Introduction

### 1.1 Background

Violence against children is a widespread problem and an unfortunate daily reality for millions of children around the world (Pinhiero, 2006; Hillis et al., 2016), also in sport (Hartill et al., 2023). Providing a uniform term and/or definition to conceptualize violence against children is difficult due to the variated terminology that is being used within literature, policy, and practice. The United Nations Convention on the Rights of the Child defines violence against children as “all forms of physical or mental violence, injury or abuse, neglect or negligent treatment, maltreatment or exploitation, including sexual abuse, while in the care of parent (s), legal guardian(s) or any other person who has the care of the child” (United Nations, 1989, art. 19). Additionally, and specifically related to sport, different conceptual frameworks define maltreatment or violence against children participating in organized sport (Stirling, 2009; Mountjoy et al., 2015, 2016; Fortier et al., 2020). The 2016 IOC consensus statement specifically refers to harassment and abuse as an umbrella terminology for all the above described forms of violence (Mountjoy et al., 2016). Therefore, throughout this article when referring to violence, abuse, maltreatment, etc., the terminology of harassment and abuse is used. Harassment and abuse constitutes out four types: psychological (e.g., threatening, shouting, or humiliating a youth sport participant), physical (e.g., shaking, punching, or hitting a youth sport participant), sexual (contact and non-contact; e.g., sexting with, masturbating with, or showing genitals to a youth sport participant), and deprivation or neglect (e.g., failing to provide sport safety equipment, refusing providing necessary medical care) (Krug et al., 2002; Mountjoy et al., 2016; Fortier et al., 2020).

Prevalence estimates of harassment and abuse in sport vary strongly (Vertommen and Parent, 2020), with recent research indicating that up to 80% of youth sport participants report at least one negative experience that could be defined as harassment and abuse in sport before the age of 18 (Parent and Vaillancourt-Morel, 2020; Willson et al., 2021; Hartill et al., 2023). Across these prevalence studies and other studies, the form of harassment and abuse that is being reported the most by youth sport participants is psychological abuse (Alexander et al., 2011; Parent and Vaillancourt-Morel, 2020; Willson et al., 2021; Hartill et al., 2023). These findings highlight that, although participation in organized sport is generally assumed to be accompanied by many benefits for the physical and psychological health of children (Biddle, 2016), and that most youth sport participants enjoy doing sport (Alexander et al., 2011), exposure to harassment and abuse in this context can jeopardize these benefits (Krug et al., 2002). Experiencing childhood harassment and abuse, either outside (Petruccelli et al., 2019) or inside of sport (Fasting et al., 2002; Krug et al., 2002; Stafford et al., 2013; Mountjoy et al., 2016; Parent and Fortier, 2018; Wilinsky and McCabe, 2020; Parent et al., 2021; Schmidt et al., 2022) is linked to poorer physical health, diminished well-being, psychological disturbances, decreased self-esteem, and social consequences. Hundreds of testimonials from (former) youth sport participants during the last decade have demonstrated similar outcomes in youth sport participants, underlining the high burden and many different adverse consequences (e.g., Fasting et al., 2002; Krug et al., 2002; Stafford et al., 2013; Mountjoy et al., 2016; Parent and Fortier, 2018; Wilinsky and McCabe, 2020; Parent et al., 2021; Schmidt et al., 2022). These negative outcomes are even greater when the child is of young age and when others do not intervene (Tillman et al., 2010; Cunnington and Clark, 2023).

Such high prevalence estimates, found consistently across countries, sport levels and disciplines, require a strategic approach to prevention in the sport sector. One avenue for the prevention of harassment and abuse is focusing on stimulating positive bystander behaviors. As such, the current study aims at systematically developing a positive bystander training program to prevent all forms of harassment and abuse in sport.

Bystanders are people who are witnesses of worrying incidents or situations, including suspected situations of harassment and abuse (Banyard et al., 2016). Bystanders are in a position where they can intervene and/or respond in and/or end situations of harassment and abuse. By doing so, they may limit the impact of violence against children. Bystander behaviors encompass both positive and negative behaviors, as well as reactive and proactive behaviors (McMahon and Banyard, 2012). Positive reactive bystander behaviors include helping someone during an incident of harassment and abuse, gaining advice when having suspicions, or reporting suspicions to the necessary authorities. Positive proactive behaviors include such as following educational programs on harassment and abuse. Unfortunately, bystanders can also perform negative bystander behaviors by doing nothing or looking away (i.e., reactive), joining in with the perpetrator (i.e., reactive), or spreading rape myths (i.e., proactive) indicating that negative bystanders behaviors are not only passive behaviors (Banyard et al., 2016). Such negative bystander behaviors are often raised in testimonials of (former) youth sport participants. Too often, bystanders did not react (adequately) to signs or incidents of harassment and abuse (Cunnington and Clark, 2023). When engaging in negative bystander behaviors, for instance, bystanders contribute to significant adverse consequences including potentially longer duration of abuse, reduced likelihood of disclosure and recovery, and reduced/ hindered access to both physical and psychological help for individuals experiencing harassment and abuse (Tillman et al., 2010; Cunnington and Clark, 2023).

Given that bystanders when engaging in positive bystander behaviors can prevent or stop harassment and abuse, it is crucially to guide and support bystanders in how to properly act before, during and after incidents of harassment and abuse. Darley and Latané (1968) highlight that in order for a person to perform positive instead of negative bystander behavior, they need to notice signs of harassment and abuse, label the incident as a problem, notice that their help is needed, and take the responsibility to take action or have the skills to do so. While these steps offer insights into the requirements for performing positive bystander behaviors, it is crucial to understand the key behavioral determinants underlying these behaviors in order to stimulate them to foster positive bystander behavior. The Reasoned Action Approach, RAA (Fishbein and Ajzen, 2010), is a theoretical model that says that people’s intentions towards a specific behavior are caused by their attitudes, norms, and beliefs regarding the behavior. More specifically, and related to positive bystander behavior, following the RAA it can be stated that the intention to perform positive bystander behaviors is determined by (A) the attitudes one has toward bystander behaviors, (B) the beliefs one has about whether relevant others approve of one engaging in bystander behavior (i.e., perceived norms), and (C) the beliefs one has about the fact that performing bystander behaviors is within their control or potential (i.e., perceived behavioral control) (Fishbein and Ajzen, 2010). Negative bystander attitudes, norms and perceived behavioral control, and a lack of knowledge, encompassing a culture of silence or tolerance for harassment and abuse (i.e., norms), lack of knowledge and understanding of what constitutes abuse, unawareness of reporting and supporting mechanisms, and fear of potential consequences are extensively described in the literature (Tillman et al., 2010; Banyard, 2011; McElvaney, 2019; Roberts et al., 2020; Cunnington and Clark, 2023) as factors that can explain the negative bystander behaviors (Tillman et al., 2010; Cunnington and Clark, 2023). All of this highlights the importance to improve and shift attitudes and norms, so that positive bystander behaviors are valued more and that it becomes the social norm (Verhelle et al., 2022).

With regard to the prevention of harassment and abuse through fostering positive bystander behaviors, most work originates from the US, targets high school students and has focused on the prevention of sexual abuse (Foubert et al., 2010; Miller et al., 2012; Katz and Moore, 2013; Coker et al., 2017; Mujal et al., 2019). A meta-analysis of Katz and Moore (2013) focused on evaluating the effectiveness of bystander programs for the prevention of sexual assault in college communities. The authors highlighted that those students following a bystander education program showed an increase in bystander efficacy, bystander behaviors, intentions to help others, and less rape myth acceptance and rape proclivity. The systematic review of Mujal et al. (2019) found similar results when evaluating sexual violence bystander intervention programs. They concluded that the use of in-person bystander interventions directed at college students can have positive effects on their bystander attitudes and self-reported bystander behaviors. Specifically, the findings underscored that the majority of the included studies consistently demonstrated favorable impact of bystander interventions on bystander attitudes, bystander efficacy, willingness to help, confidence to help, and a decrease in rape myth acceptance. Though both reviews shed a positive light on the use of bystander intervention in the prevention of harassment and abuse, both indicate specific limitations which focus on the limited number of available studies, limited statistical power, questionable practical effects of the attitudinal changes within the studies, and they highlight the need for longitudinal evaluations (Katz and Moore, 2013; Mujal et al., 2019).

Looking at specific programs, an example of a bystander program is the Green Dot violence prevention program which is a theory-based bystander program training high school students to recognize situations of violence (Coker et al., 2017). The program has been developed to reduce sexual violence and related forms of interpersonal violence by promoting positive bystander behaviors among high school students. Methods used in the program were the popular opinion leader strategy and individual feedback. Implementation and evaluation of the program, using a randomized controlled trial, showed a long-term significant decrease in sexual violence perpetration and victimization. Though the program focuses on bystander behaviors, the outcomes of this study did not focus on bystander behaviors, rather on the presence of violence perpetration and victimization. Additionally, using a randomized controlled trial for a complex phenomenon as changing behavioral determinants might not be the most feasible option as it is questionable if the results are representative for the real-life context that is more complex compared to an experimental set-up. Another program that has been developed specifically for college women, is a rape awareness program called the Women’s Program (Foubert et al., 2010). This program has been designed to empower college women to engage in positive bystander behaviors in potential rape situations by providing them with theory and discussions on how to help a friend during and after dangerous situations. The results of an experimental study showed a decrease in rape myths acceptance, an increase in confidence in ability to intervene, and perceived willingness to help. Nevertheless, an important limitation of this study is that the observed attitudinal changes are not assessed for their long-term effects, indicated that it is not sure if these changes remain present and that the participants will continue to perform positive bystander behaviors. A third program within a high school context that has been studied extensively is the Coaching Boys Into Men (CBIM) program (Miller et al., 2012). This program has been set up as an easy-to-implement program, implemented by athletic coaches in high school contexts and focused on altering youth sport participants’ norms about dating violence. Athletic coaches were trained to facilitate brief team discussions during their practice by using predefined conversation topics related to violence prevention. These discussions are used with the intention to improve youth sport participants’ attitudes and behaviors regarding dating violence (Miller et al., 2012). The results of a randomized controlled trial showed an increase in recognition of abusive behaviors and intentions to intervene, and an increase in positive bystander behaviors (Miller et al., 2012). These interventions adapt to fostering positive bystander behaviors among college students (Foubert et al., 2010; Coker et al., 2017) and high school athletic coaches (Miller et al., 2012) show promising results in terms of preventing sexual or dating violence. Yet, up until today no interventions have been developed that can be implemented at the level of the sport club hereby targeting positive bystander behaviors in relation to all four types of harassment and abuse. To our knowledge, only two bystander interventions have been implemented and evaluated within the specific context of the sport club, both focusing on sexual violence (Schäfer-Pels et al., 2023). Verhelle et al. (2024) developed a theory-driven bystander intervention, called All Aboard, specifically targeting youth sport coaches in Flanders. The main aim of the All Aboard intervention was to stimulate positive bystander behaviors among youth sport coaches in relation to adequately detecting, assessing, and responding to signs of sexual harassment and abuse. Different strategies were used including theory, watching testimonials and discussions. Results showed that after the intervention, youth sport coaches showed more positive attitudes related to positive bystander behaviors (Verhelle et al., 2024). The main limitation of this study was the limited sample size, single focus on sexual harassment and abuse, focus on only one target group, and high drop-out rates. Schäfer-Pels et al. (2023) developed an intervention, called Qualifizierungsmodul “Gegen sexualisierte Gewalt im Sport “or Module Against Sexualized Violence in Sport, to act against sexual violence in sport by focusing on raising awareness, prevention and handling cases of potential harassment and abuse. The module consisted of a sensitizing workshop focusing on knowledge of sexual violence, personal attitudes towards the prevention of sexual violence, intentions to act against sexual violence, and preventive measures against sexual violence. The methods used included awareness raising and discussions. The module was available for coaches, youth sport participants, board members, supervisors of the team, and parents. The results of the study indicated positive short- and long-term effects on attitudes, perceived behavioral control, intentions to act, and knowledge (Schäfer-Pels et al., 2023). Although the results were promising, and the authors focus on both coaches, youth sport participants, board members, supervisors of the team, and parents, it is not clear what the benefits could be of tailor-made modules.

Both the All Aboard program (Verhelle et al., 2024) and the Module Against Sexualized Violence in Sport (Schäfer-Pels et al., 2023) showed some first promising results in stimulating determinants that are at the basis of positive bystander behaviors to prevent sexual harassment and abuse in sport. These programs can serve as a starting point to build upon to develop more extensive bystander programs that focus on all types of harassment and abuse. Indeed, acknowledging the high prevalence of other non-sexual forms of harassment and abuse and often co-occurring experiences of psychological, physical, sexual harassment and abuse and/or neglect, a broader focus is recommended. Additionally, it is important to acknowledge that everyone in a sport club can be a witness of an incident of harassment and abuse (Banyard et al., 2016). The previous mentioned studies outside (Foubert et al., 2010; Miller et al., 2012; Katz and Moore, 2013; Coker et al., 2017; Mujal et al., 2019) and inside sport (Verhelle et al., 2024) solely focused on one target group. In contrast, the module of Schäfer-Pels et al. (2023), expanded their module to also coaches, parents, and so on. This indicates the need and importance to include different target groups and to develop tailored-made programs to foster positive bystander behaviors within the sport club when focusing on the prevention of harassment and abuse.

### 1.2 Present study

The objective of the current paper is to describe the theory-driven development of the Safe Sport Allies bystander training program for youth sport participants and youth sport coaches, and to describe future research plans that include the implementation and evaluation of the program. While many people in grassroots sport clubs (e.g., parents, club administrators, referees, …) can act as positive bystanders, we focus in the current study on youth sport participants and their coaches. The process of developing such interventions is complex and challenging, therefore the Intervention Mapping approach (Bartholomew et al., 1998, 2016) was used. The Intervention Mapping approach serves as a comprehensive guide for the development and planning of health promotion programs, offering a structured framework with distinct and clear steps (Bartholomew et al., 1998). The approach consists of six steps: (1) logic model of the problem, (2) program outcomes and objectives, (3) program design, (4) program production, (5) program implementation plan, and (6) evaluation plan (Bartholomew et al., 2016). In what follows, the different steps of the Intervention Mapping approach will be explained and applied to the development of an intervention to promote positive bystander behaviors among sport participants and sport coaches (i.e., Safe Sport Allies) within the grassroot sport club context.

## 2 Methods

Based on the undeniable issue of harassment and abuse, the discussed bystander programs available outside and inside sport, and how these programs often only target one target group, the Safe Sport Allies consortium aimed at systematically developing tailor-made bystander training programs for youth sport participants and youth sport coaches to be implemented in local sport clubs. Safe Sport Allies is an international collaborative partnership, co-funded by Erasmus+ (622589-EPP-1-2020-1-BE-SPO-SCP), between two sport organizations (Cyprus Sport Organization, CY; Athletic Club Foundation Bilbao, ES), three institutes of higher education (Thomas More University of Applied Sciences, BE; Open University, NL, Haaga-Helia University of Applied Sciences, FI) and one research center (Mulier Institute, NL), one international child protection agency (Terre des Hommes, RO), one safe sport practice developer (Center Ethics in Sport, BE) and one survivor led organization (De Stilte Verbroken, NL). These organizations have a complementary background, experience, and expertise in safeguarding policies in and outside sport. The objectives of the Safe Sport Allies partnership and project was fourfold: (1) developing bystander interventions for youth sport participants, youth sport coaches, and parents, and developing a policy and implementation trajectory for club administrators, (2) developing a measurement toolkit for the monitoring and evaluation of the interventions, (3) longitudinal testing the effectiveness of the bystander interventions for youth sport participants and coaches, and (4) disseminating the findings and Safe Sport Allies materials. All partners (including the authors of the current paper) within the consortium worked together during the different phases of the project and development, implementation and evaluation of the Safe Sport Allies bystander training program from January 2021 until June 2023. The current paper focuses on how the Intervention Mapping approach was applied to the development of the bystander training program for youth sport participants and youth coaches.

### 2.1 Step 1 intervention mapping: logic model of the problem

**Figure 1:**
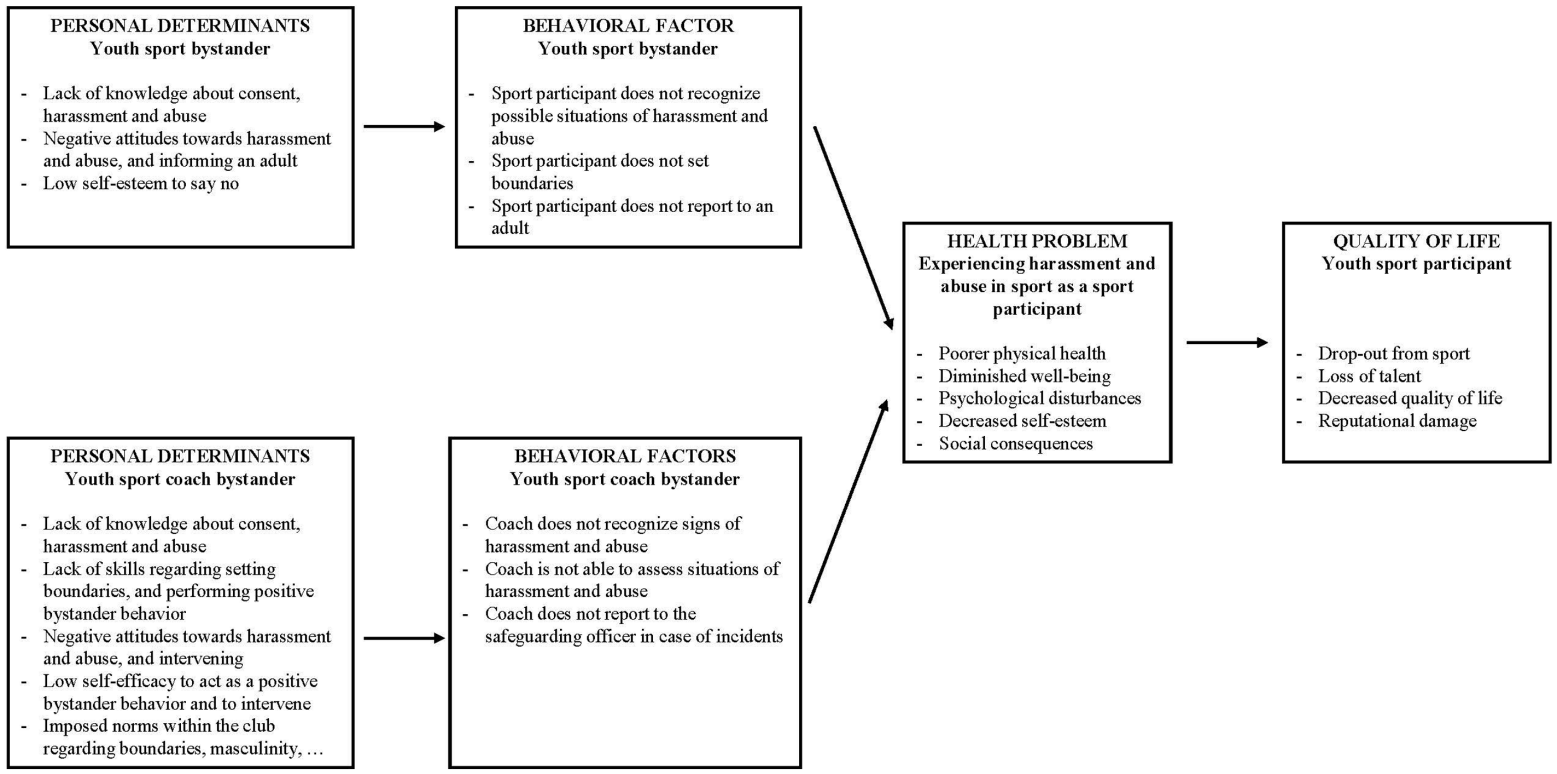
Logic model of the problem.

The first step of the Intervention Mapping approach is to establish a *needs assessment* of the health problem by identifying what needs to be addressed and for whom, which results in a logic model of the problem (see Figure 1) (Bartholomew et al., 2016). Based on the literature overview presented in the introduction, the needs assessment focused on the target groups of youth sport participants and youth sport coaches. In addition to this literature review, the partners in the consortium completed a mapping regarding the currently available prevalence studies of harassment and abuse, legal and policy frameworks, and an overview of the current educational, safeguarding and bystander intervention programs available in their country. This mapping resulted in a safe sport comparative analysis in seven countries (Stevens et al., n.d.). Additionally, interviews were carried out with survivors and bystanders to gain more insights into their needs when it comes to safeguarding (Stevens, n.d.). Based on the literature review, country mapping, and the interviews, the needs assessment indicates that the current health problem encompasses youth sport participants’ exposure and victimization of harassment and abuse in sport (Krug et al., 2002; Mountjoy et al., 2016; Fortier et al., 2020; Hartill et al., 2023), see Supplementary Table S1. As opposed to other health problems (e.g., smoking, alcohol abuse), the consequences of harassment and abuse felt by the victim are caused by another individual displaying harmful behavior. In this case, the health problem is initiated and/or maintained by perpetrators and/or bystanders who lack intervening in cases of harassment and abuse. The problem of bystanders not intervening can be related to their lack of noticing signs of harassment and abuse, being unable to judge the signs or incident, not labeling the incident as a problem, not noticing that their help is needed, being in doubt about what to do, not feeling confident to respond, and not taking the responsibility to take action or have the skills to do so (Darley and Latané, 1968; Fenton and Mott, 2018; Spaaij and Schaillée, 2019).

Finally, using the results from the needs assessment, Step 1 of the Intervention Mapping approach ends with stating the *program goals*. Based on the health problem of youth sport participants being exposed to harassment and abuse in sport, the many negative consequences this has, and its accompanying environmental risks, the current program goal can be defined as stimulating positive bystander behaviors among youth sport participants and youth sport coaches in grassroots sport clubs. Changes will be measurable at the level of the different individual behavioral determinants that will be touched upon during the different programs (see further).

### 2.2 Step 2 intervention mapping: program outcomes and objectives, and logic model of change

Step 2 of the Intervention Mapping approach focuses on which determinants need to be changed to improve the health problem (Bartholomew et al., 2016). In this case, the focus in Step 2 lies on determining which behavioral determinants must be changed among youth sport participants and youth sport coaches to stimulate positive bystander behaviors (i.e., behavioral outcome). Therefore, the bystander training programs focuses on stimulating and increasing positive bystander behaviors by targeting key behavioral determinants related to this behavior defined by the reasoned action approach (Fishbein and Ajzen, 2010): (A) the attitudes one has toward bystander behaviors, (B) the beliefs one has about whether relevant others approve of one engaging in bystander behavior (i.e., perceived norms), and (C) the beliefs one has about the fact that performing bystander behaviors is within their control or potential (i.e., perceived behavioral control) (Fishbein and Ajzen, 2010).

**Figure 2:**
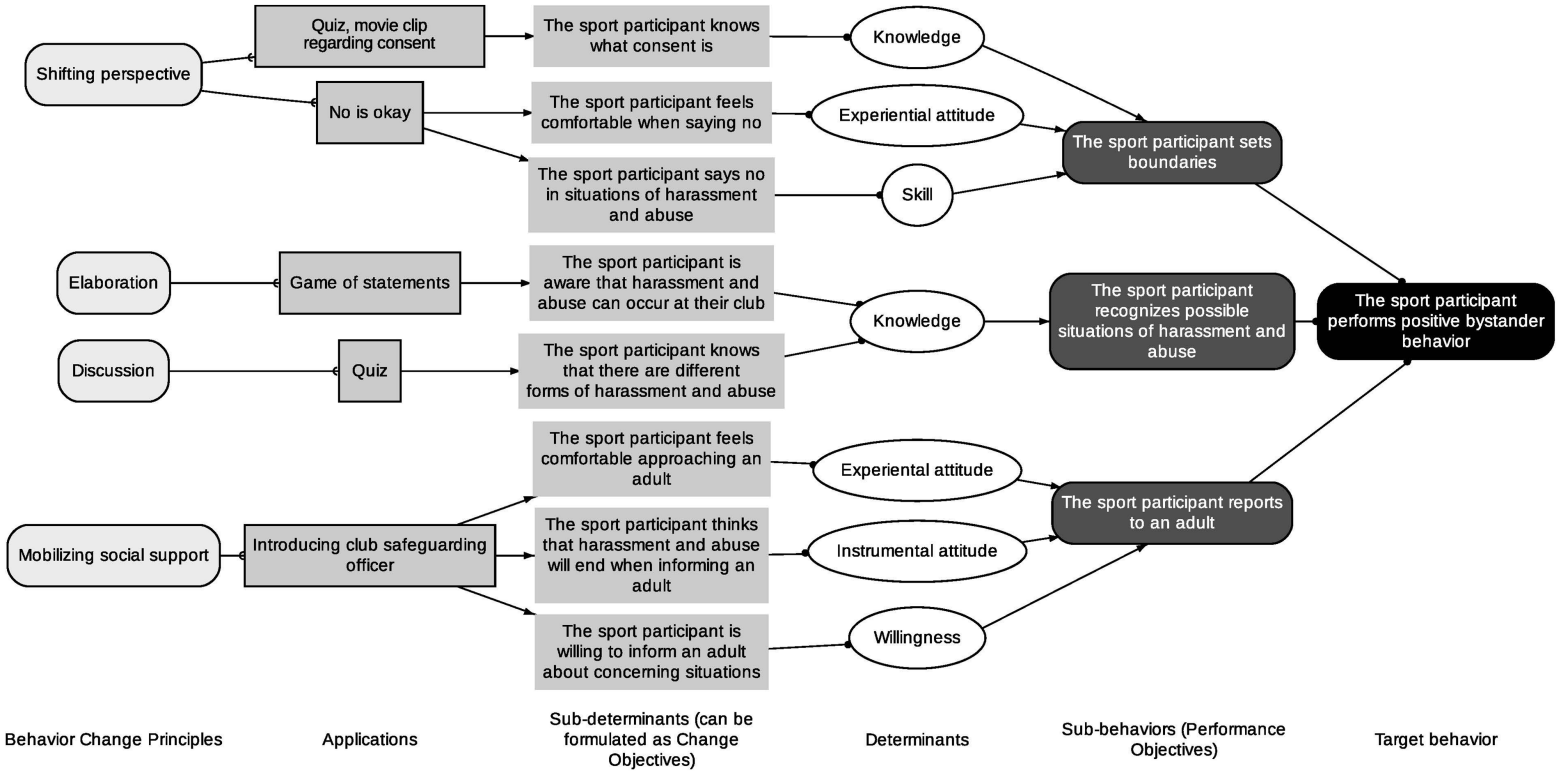
Overview application intervention mapping to the safe sport allies bystander training program for youth sport participants, steps 1 to 3.

**Figure 3:**
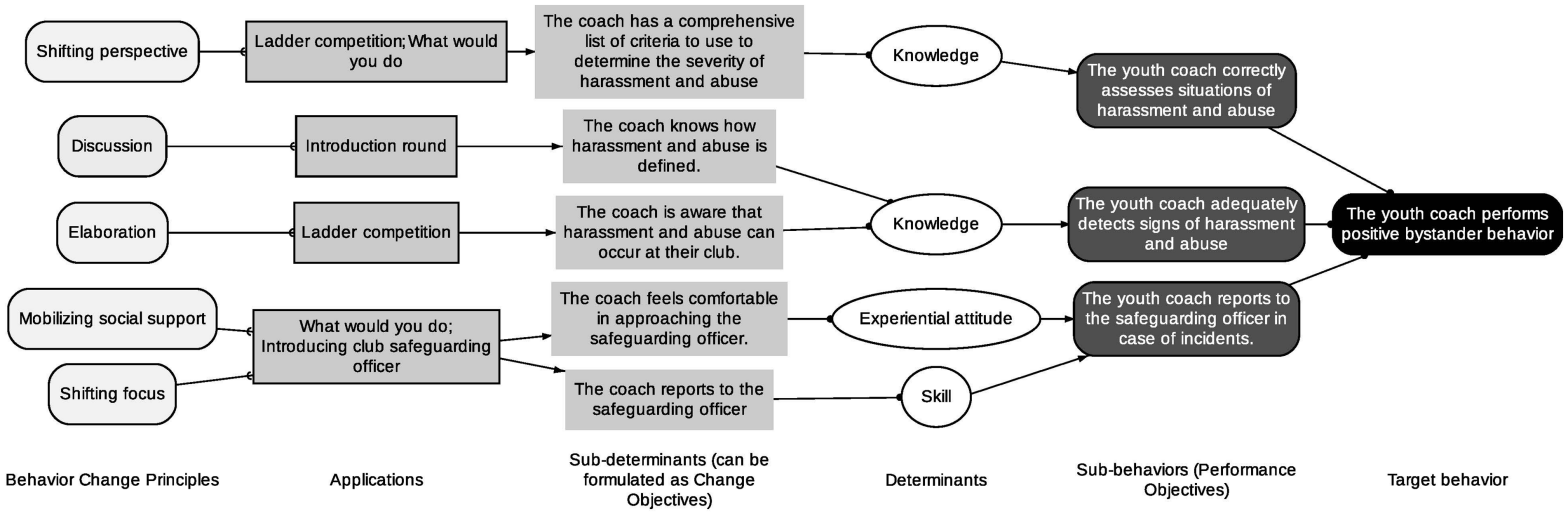
Overview application intervention mapping to the safe sport allies bystander training program for youth sport coaches, step 1 to 3.

The overall behavioral outcome for each of the different programs and therefore for both target groups is the same, more specifically performing positive bystander behaviors. However, the specific performance objectives of each of the target groups differ. Performance objectives are specified actions and behaviors that must be taken to achieve the behavioral outcome (Bartholomew et al., 2016). Subsequently, all performance objectives are linked to specific change objectives that represent specific behavioral determinants. All details regarding the health problem, performance objectives, and change objectives for each of the target groups are presented in Figures 2, 3 and in Supplementary Table S1.

### 2.3 Step 3 intervention mapping: program design

Next, the program design was developed which firstly focuses on generating the *intervention themes* (Bartholomew et al., 2016), which for the current intervention was stimulating positive bystander behaviors among youth sport participants and youth sport coaches. In order to develop the program, the mapping (see Step 1 Intervention Mapping) across countries was used to inventory current available bystander programs which could be adapted for the current bystander training programs. Regarding the training programs, subthemes such as setting and respecting boundaries, consent, assessing situations of harassment and abuse, and learning where to report were included.

Secondly, the *change methods* used include discussion, elaboration, shifting perspective, and mobilizing social support (Bartholomew et al., 2016). These methods were chosen as they have been used previously (Foubert et al., 2010; Miller et al., 2012; Coker et al., 2017; Schäfer-Pels et al., 2023; Verhelle et al., 2024), but also have a theory-based foundation in increasing knowledge, changing attitudes, beliefs, and outcome expectations, changing social norms and social influences which are the main behavioral determinants upon which the training program will focus. In the next step, the different themes and methods were paired with appropriate practical applications. Some examples of practical applications include a quiz, video, introduction safeguarding officer, etc., see Figures 2, 3 and Supplementary Table S1 for all the details.

### 2.4 Step 4 intervention mapping: program production

The chosen methods and practical applications guided the development process of the *program materials* for the different bystander training programs. Table 1 provides an overview of all the details of the two bystander training programs. Additionally, detailed information regarding the content of the programs can be found on the project’s website www.safesportallies.eu. Because it is well-established that adolescence is an important period (and also opportunity) for youth to develop specific skills, values, attitudes that are needed for their own well-being but also for their social development and social interaction with others later on in life (Bornstein et al., 2012), the bystander training program for *youth sport participants* targets 12- to 14-year-olds. The bystander training program is designed to be implemented in groups of eight to 12 youth sport participants to allow for sufficient peer interactions. The program includes a one-time in-person workshop of 90 min that focuses on learning about setting and respecting boundaries, consent, and where to report (i.e., getting acquainted with the club safeguarding officer).

**Table 1:** Overview bystander training program, aims, and components.

Target group	Bystander training program	AIMS	Components
Youth sport participants (12-14 years)	Face-to-face workshop (90 minutes)	Learning to set and respect personal boundaries Learning how to recognize situations of harassment and abuse Knowing where to report	Based in part on ‘Child sexual abuse stays offside’ (Spanish High Council for Sport, 2018) and ‘Sport op jongerenmaat’ (Centrum Ethiek in de Sport, 2021) Exercises that stimulate interaction and discussion Cases of physical, psychological, and sexual harassment and abuse, and neglect IOC Consent in Sport video (IOC Athlete 365, n.d.)
Youth sport coaches	Face-to-face/online workshop (120 minutes) Three online booster sessions	Learning to recognize signs of harassment and abuse Learning to assess situations of harassment and abuse Knowing where to report	Exercises that stimulate interaction and discussion Examples and cases of physical, psychological, and sexual harassment and abuse, and neglect

The bystander training program for youth sport coaches includes a one-time in-person or online (upon request of the sport club) workshop of 120 min and three online booster sessions that follow in the three consecutive weeks after the actual workshop. Based on the experiences of previous research from Verhelle et al. (2024) the bystander training program is designed to be implemented in groups of five to eight youth sport coaches. During the workshop, the youth sport coaches learn to recognize signs of harassment and abuse, and how to assess and react to such situations. Like the intervention for youth sport participants, youth sport coaches got to know the club safeguarding officer. The idea is that the safeguarding officer of the club is invited to the workshop and that they can explain their role, in order that both youth sport participants and youth sport coaches get acquainted with the person and their role. The additional online booster sessions included movie clips (IOC Athlete 365, n.d.; NOC*NSF, n.d.) and a scenario-based exercise (for an example see the Supplementary materials, section 2). These online booster sessions are included because the brief literature review on bystander interventions, from the Safe Sport Allies consortium, highlighted that increasing the ‘dosage’ of the intervention or program content can enhance positive outcomes, in this case further improving positive bystander behaviors (Stevens, 2022).

### 2.5 Step 5 intervention mapping: program implementation plan

Step 5 of the Intervention Mapping approach includes the development of the implementation plan to enable the implementation of the Safe Sport Allies bystander training program. The Safe Sport Allies bystander training program is developed to be implemented in grassroots sport clubs.

The implementation plan consists of the following steps: (1) distributing a call for participation using a flyer, (2) signing up of clubs for the whole program or specific modules, and (3) organizing workshops for youth sport participants or coaches.

The youth sport participants bystander training program is developed for 12 to 14-years-olds. The module for youth sport coaches aims at coaches of at least 18 years old with a coaching experience of at least 6 months. No other specific criteria need to be considered when implementing these modules.

### 2.6 Step 6 intervention mapping: evaluation plan

#### 2.6.1 Research design

The developed evaluation plan of the bystander training programs for youth sport participants and youth sport coaches includes a longitudinal quasi-experimental design. The bystander training program for youth sport participants is developed in such a way that they can be evaluated using pre and post measurements (T0, T1). Because the program for youth sport coaches also includes booster sessions after the in-person or online workshop, the intervention can be longitudinally evaluated by comparing pre-, post-, and follow-up measurements (T0, T1, T2).

#### 2.6.2 Questionnaires

To evaluate the different bystander training programs, it is suggested to use questionnaires that focus on the different key behavioral determinants that the programs try to target upon. For youth sport participants the developed questionnaire that can be administrated consists of four sections (see Table 2 for all details): (1) basic sociodemographic variables, (2) knowledge of harassment and abuse, (3) behavioral determinants such as knowledge, perceived norms, instrumental attitudes, and intentions toward consent, setting and respecting boundaries, and reporting, and (4) a slightly adapted version of the Student Bystander Behavior Scale (Thornberg and Jungert, 2013), and (5) user feedback.

**Table 2:** Sections and content questionnaire youth sport participants.

Section	Content questions
Sociodemographics	Two items – Open-ended question and predefined categories Age Gender
Knowledge harassment and abuse	Two items – Open-ended questions Understanding – definition Examples
Behavioral determinants	Five items – 5-point Likert scale; ranging from totally disagree – totally agree, e.g., *‘Consent is important when it comes to indicating your own boundaries’* ‘*I intend to inform an adult when someone crosses my boundaries’*
Student Bystander Behavior Scale (Thornberg and Jungert, 2013)	Eight items – 5-point Likert scales ranging from definitely not – definitely yes Focus on different types of bystander behavior: defender, outsider, pro-bully behaviors
User feedback (only at T1)	Four items – 7-point Likert scales: Interesting Learned Recommend Difficulty level

The questionnaire for the evaluation of the bystander training program for youth sport coaches can include an assessment of the (1) basic sociodemographic variables, (2) knowledge of harassment and abuse, (3) Readiness to change scale (Banyard et al., 2009) adapted to the Flemish sport context, (4) behavioral determinants such as youth sport coaches’ knowledge, attitudes, perceived behavioral control, intentions, and current behavior (i.e., past behavior and intention) towards consent, setting and respecting boundaries, noticing signs of harassment and abuse, and reporting when concerned, (5) the adapted Student Bystander Behavior Scale (Thornberg and Jungert, 2013) (see Table 3)

**Table 3:** Sections and content questionnaire youth sport coaches.

Section	Content questions
Sociodemographics	Seven items – Open-ended and predefined questions Age Gender Sport club Reason for participation Expectations of the intervention Coaching experience Current coaching career
Knowledge harassment and abuse	Two items – Open-ended questions Understanding – definition Examples
Readiness to change scale (Banyard et al., 2009)	Six items – Likert scales with varying anchor points Adapted to the Flemish sport context, e.g., ‘*I can do something about harassment and abuse in my sport club*’ ‘*I intend to figure out what I can do against harassment and abuse*’
Behavioral determinants	Sixteen items – Likert scales with varying anchor points, e.g., ‘*I listen to my youth sport participants when they indicate their boundaries*’ ‘*When I intervene in situations of harassment and abuse, the situation will worsen*’ ‘*I intend to ask consent from my youth sport participants when I want to touch them at the arm or shoulder in order to perform a movement*’
Student Bystander Behavior Scale (Thornberg and Jungert, 2013)	Eight items – 5-point Likert scales ranging from definitely not – definitely yes Focus on different types of bystander behavior: defender, outsider, pro-bully behaviors
User feedback (only at T1 and T2)	Six items – Likert scales with varying anchor points Interesting, learned, recommend, difficulty level Value different parts of the workshop Interesting and value online boosters

#### 2.6.3 Analysis plan

The developed analysis plan for the evaluation of the bystander training programs for youth sport participants and coaches is similar. For both programs, the main aim is to evaluate the programs by assessing whether the programs result in a positive change regarding the behavioral determinants that influence the intentions and positive bystander behaviors of these target groups. Firstly, descriptive analyses (frequencies, proportions, and means) will be used to analyze the sociodemographic variables of youth sport coaches and youth sport participants, as well as for the youth sport coach coaching related variables (e.g., coaching experience). Exploratory qualitative analyses will be used to analyze the open-ended knowledge questions focusing on the definition and examples of harassment and abuse.

Next, a bystander index will be calculated based on the responses to the statements that focus on the different behavioral determinants that influence positive bystander behavior. For youth sport participants, the statements focus on their knowledge, perceived norms, instrumental attitudes, and intentions towards consent, setting and respecting boundaries, and reporting when concerned. For youth sport coaches, these include statements on their knowledge, attitudes, perceived behavioral control, intentions, and current behavior towards consent, setting and respecting boundaries, noticing signs of harassment and abuse, and reporting. Additionally, for each of the behavioral determinants and objectives of the intervention, a subscale is calculated.

To evaluate the changes over time after having participated in the workshops, general linear models will be used that include the T0 and T1 data for the youth sport participants and coaches and subsequently also including T2 data for the coaches. Similar analyzes are conducted for the data from the Readiness to change and Student Bystander Behavior scale. Lastly, descriptive analyzes are used for the user feedback from youth sport participants and coaches.

## 3 Discussion

To our knowledge, while harassment and abuse is a clear problem in grassroot sport clubs (Fasting et al., 2002; Krug et al., 2002; Mountjoy et al., 2016; Parent et al., 2021; Schmidt et al., 2022), limited positive bystander programs have been systematically developed and tested for the prevention of harassment and abuse in grassroots sport clubs. Nevertheless, bystander programs in sport are available, such as the bystander empowerment program developed by Coaching Association of Canada (n.d.), though it is not clear to what extend these programs are evaluated and tested. The Safe Sport Allies Erasmus+ collaborative partnership aimed at developing, implementing, and testing tailor-made bystander training programs for youth sport participants and youth sport coaches. The current paper describes the application of the Intervention Mapping approach for the bystander training program for youth sport participants and youth sport coaches. The Safe Sport Allies bystander training program was developed from a holistic point of view (Banyard, 2011; Roberts et al., 2020) using the Intervention Mapping approach (Bartholomew et al., 2016), and the Reasoned Action Approach (Fishbein and Ajzen, 2010), as a scientific foundation. In the current paper, we described the different steps of the Intervention Mapping approach and how we have applied these steps to the Safe Sport Allies bystander training program. The training program was presented here, including all causal-structural assumption chains underlying its hypothesized effectiveness as an acyclic behavior change diagram, as well as in machine-readable ABCD matrices (Metz et al., 2022). These provide insight into the putative mechanisms of action and facilitate adoption of the intervention to different contexts.

The novelty of the Safe Sport Allies bystander training program is twofold. On the one hand, the bystander training programs have been tailor-made for specific target groups. After a thorough needs assessment, the health problem (i.e., exposure to harassment and abuse by youth sport participants) and the specific program goals were described for youth sport participants and youth sport coaches, while the performance and change objectives have been described separately for each group to match their needs. On the other hand, the Reasoned Action Approach has been used as a guiding theory to disentangle the health problem and the target behavior (i.e., performing positive bystander behaviors) to specific behavioral determinants at the individual level upon which the bystander training program should focus.

The training programs have been developed in such a way that they are ready to be implemented in grassroots sport clubs. Nevertheless, the program can also be considered for other applications as well, such as elite sport, youth work, and youth academies. Important here is to consider a reassessment of the needs assessment, as it is possible that new settings might indicate or suggest necessary changes to the current program if one wants to implement the program in, for example, an elite sport context. The Intervention Mapping approach includes ways to use this approach to adapt evidence-based interventions (Bartholomew et al., 2016).

Regarding the proposed implementation and evaluation plans, a main objective is to evaluate whether the intervention improves or changes the different behavioral determinants that were targeted during the program. The focus is not to evaluate the theory that is used for the development of the program, but evaluate the changes in behavioral determinants, such as knowledge, attitudes, norms, and perceived behavioral controls. Questionnaires have been developed that focus on these behavioral determinants that are related to positive bystander behaviors. Important here is to acknowledge that actual positive bystander behaviors cannot be assessed, and that only the underlying behavioral determinants will be measured as a proxy for positive bystander behavior. Additionally, previous research (Miller et al., 2012; Schäfer-Pels et al., 2023) mostly evaluated bystander interventions in the short-term, the current program and proposed evaluation tools allow for longitudinally assess changes regarding behavioral bystander determinants, especially for sport participants and youth sport coaches.

Although the Safe Sport Allies bystander training program and the way it has been developed can be a starting point for the further development of such programs in sport, it is important to consider the following limitations, especially in context of future research. The presented bystander training program focused mainly on the individual determinants related to positive bystander behaviors, however, it is important to acknowledge that incidents of harassment and abuse do not occur in a vacuum and that there is a need to develop a whole-system approach (Roberts et al., 2020). A whole-system approach can be seen as an inclusive and systematic approach in which different target groups are included and targeted. By establishing a whole-system change, the different environmental (risk) factors can positively be influenced (Mountjoy et al., 2016; Roberts et al., 2020) both at an individual but also at the club policy level. The different environmental risks at the interpersonal, organizational, community, and societal level may thus also impact bystander behaviors (Bartholomew et al., 2016). When looking at environmental risks, Roberts et al. (2020) highlighted two main organizational factors that can be seen as a catalyst for all forms of harassment and abuse, more specifically organizational tolerance and conformity to dominant values. Organizational tolerance for harassment and abuse can lead the way for the occurrence of it as it implies that perpetrators will not be punished, that notifiers most likely experience some form of repercussion, that bystanders should stay passive, and that the definition of harassment and abuse is not clear in the organization. When such organizational tolerance is displayed in these dominant values, norms and beliefs, this will result in a continuous and reinforcing process of the occurrence of harassment and abuse in sport (Roberts et al., 2020). For future research, it is suggested to also include the community and societal levels when establishing a whole-system change.

In addition to the presented bystander training programs in this paper, the Safe Sport Allies Erasmus+ project also focused on sport parents and club administrators. In future research, it would be interesting to include these levels (and additional levels such as officials) as well and to systematically develop the bystander training programs for these groups as well. Although in the overall project, for example, a policy trajectory for club administrators was developed which focused on establishing changes in policies, it was not based on a systematic needs assessment. It is thus recommended to carry out a new cycle of the Intervention Mapping approach, or use Intervention Mapping as a way to adapt evidence-based interventions (Bartholomew et al., 1998, 2016). The same is true for other important target groups that are around the youth sport participants, such as parents or officials.

Furthermore, it is important to highlight that throughout the development of the Safe Sport Allies bystander training program, feedback has been gathered from different target groups and experts to improve the content of the program. The Intervention Mapping approach allows for adjusting the interventions during the development process when needed (Bartholomew et al., 2016). Secondly, in step 6 of the Intervention Mapping approach it is described how self-reported questionnaires can be used to evaluate the changes regarding the behavioral determinants related to performing positive bystander behaviors. For future research, it is imperative to consider additional methodologies to evaluate the programs, such as using mixed methods. Finally, a challenge for the implementation of the Safe Sport Allies program is the lack of awareness of the importance of preventing harassment and abuse in sport among club administrators and those involved in the club. Raising awareness among these clubs is of importance to minimize thresholds that might be related to unfamiliarity or ignorance of the problem. Clubs also need to be supported when thresholds related to capacity and resources are observed when implementing the program.

## 4 Conclusion

Experiences of harassment and abuse are frequently reported by sport participants in grassroot clubs. Such experiences can be prevented if people within the sport club display positive bystander behaviors such as noticing signs of harassment and abuse, helping others when noticing incidents, or going to the safeguarding officer when having concerns. The current paper delved into the comprehensive development process of the a positive bystander intervention program (i.e., Safe Sport Allies) for youth sport participants and youth sport coaches that can be implemented in grassroots sport clubs to prevent all types of harassment and abuse. The program, and implementation and evaluation plans described in this paper can serve as a starting point and source to build future interventions within this critical domain of safeguarding in (and outside) sport.

## Data availability statement

The original contributions presented in the study are included in the article/Supplementary material, further inquiries can be directed to the corresponding author.

## Author contributions

KA: Conceptualization, Investigation, Methodology, Project administration, Visualization, Writing – original draft, Writing – review & editing. HV: Conceptualization, Funding acquisition, Investigation, Methodology, Project administration, Writing – review & editing. G-JP: Writing – review & editing, Conceptualization, Methodology. LH: Writing – review & editing. TV: Conceptualization, Funding acquisition, Investigation, Methodology, Project administration, Supervision, Writing – review & editing.
