# Supplementary material for: The Safe Sport Allies bystander training: developing a multi-layered program for youth sport participants and their coaches to prevent harassment and abuse in local sport clubs
Source: Front Psychol. 2024 Jun 19;15:1389280. doi: 10.3389/fpsyg.2024.1389280 (PMC11223523; doi:10.3389/fpsyg.2024.1389280)
Supplement: Supplementary file 1 [file Table_1.DOCX]

Supplementary Material

# Tables

Table A

*Overview application intervention mapping approach to the Safe Sport Allies bystander training program for youth sport participants and youth sport coaches, steps 1 to 3*

| **IM step** | **Step 1: needs assessment** | | **Step 2: program outcomes and objectives** | | **Step 3: program design** | |
| --- | --- | --- | --- | --- | --- | --- |
| **IM terms** | ***Quality of life problem***  Define the health or quality of life problem | ***Program goals***  Define the program goals | ***Performance objectives***  Identify behaviors that can help reduce the health problem | ***Change objectives***  Select determinants that influence these behaviors | ***Change methods***  Theoretical methods chosen to influence the change objective | ***Applications or strategies***  Methods matched with practical application |
| Applied to the Safe Sport Allies educational program for sport participants, youth coaches, and sport parents | Exposure to harassment and abuse by young sport participants | 1. Stimulating positive bystander behaviors among youth sport participants and youth coaches | **YOUTH SPORT PARTICIPANTS** | | | |
|  |  |  | 1. The sport participant recognizes possible situations of harassment and abuse 2. The sport participant sets boundaries 3. The sport participant reports to an adult | **CO1.1** The sport participant knows that there are different forms of harassment and abuse  **CO1.2** The sport participant is aware that harassment and abuse can occur at their club  **CO2.1** The sport participant knows what consent is  **CO2.2** The sport participant feels comfortable when saying no  **CO2.3** The sport participant says no in situations of harassment and abuse  **CO3.1** The sport participant feels comfortable approaching an adult  **CO3.2** The sport participant thinks that harassment and abuse will end when informing an adult  **CO3.3** The sport participant is willing to inform an adult about concerning situations | Discussion (increasing knowledge via an open informal debate)  Elaboration (increasing knowledge and changing attitudes, beliefs, and outcome expectations by adding meaning to the processed information)  Shifting perspective (changing attitudes, beliefs, and outcome expectations by being encouraged to take the perspective of the other)  Mobilizing social support (changing social influence by prompting communication about behavior change) | Quiz  Game of statements  Quiz – Movie clip ‘Consent’ (IOC)  No is okay  Introduction safeguarding officer |
|  |  |  | **YOUTH SPORT COACHES** | | | |
|  |  |  | 1. The youth coach adequately detects signs of harassment and abuse 2. The youth coach correctly assesses situations of harassment and abuse 3. The youth coach reports to the safeguarding officer in case of incidents. | **CO1**.**1** The coach knows how harassment and abuse is defined.  **CO1.2** The coach is aware that harassment and abuse can occur at their club.  **CO2.1** The coach has a comprehensive list of criteria to use to determine the severity of harassment and abuse  **CO3.1** The coach feels comfortable in approaching the safeguarding officer.  **CO3.2** The coach reports to the safeguarding officer | Discussion  Elaboration  Shifting perspective  Mobilizing social support  Shifting focus | Introduction round  Ladder competition  Ladder competition & What would you do  What would you do & Introduction safeguarding officer |

# Program materials – Booster session youth sport coaches

Below an example of a scenario-based exercise is described which was included in the online booster sessions for youth coaches. Scenario-based exercises are available on <https://www.grenswijs.be/>.

*Locked in the restroom*

Since the start of the school year, 18-year-old gymnast Samira has endured profound loneliness due to the actions of several of her team mates at the elite sports boarding school. They have engaged in a series of cruel behaviors, including hiding her clothes, mocking her whenever she makes a mistake during exercises, and deliberately excluding her from social activities. Upon entering the gym, their derisive laughter and pointed glances only intensify her distress.

Their actions escalate when they cut a portion of Samira's hair while she is asleep in the boarding school and, shockingly, lock her inside the restroom. When the boarding school counselor becomes aware of the situation, Samira courageously confides in him. Recognizing the severity of the issue, he promptly notifies the school welfare officer.
